# Bee Stings and Chronic Pain Disorder

**DOI:** 10.7759/cureus.2060

**Published:** 2018-01-13

**Authors:** Peter Steffen, Hannes Hofbauer, Michael Noll-Hussong

**Affiliations:** 1 Department of Anesthesiology, University Hospital Ulm; 2 Department of Psychosomatic Medicine and Psychotherapy, Saarland University

**Keywords:** chronic pain, psychiatry, pain management, bee, headache, mental health, psycho- social behaviour, psychosomatic medicine, complementary and alternative medicine, placebo

## Abstract

Chronic pain is a major problem of public health, and patients seek manifold forms of help to find relief. Here we present and discuss the case of a middle-aged woman suffering from mental disorders who treated her headache with the self-application of bee stings on her back.

## Introduction

Chronic pain is a major problem of public health worldwide and is jointly responsible for an increase in health costs. The therapeutic options available for the treatment of chronic pain are often limited and unsatisfactory, accompanied by a high number of adverse reactions, tolerance, and dependence, and can also reduce the quality of life, (pharmaco)therapy adherence, and functional capacity [1]. Patients often seek help for their ongoing pain, and the means that they subjectively find helpful or pain-relieving could be surprising, one-sided, dangerous, or part of a psychopathology on its own. Here we present the case of a middle-aged woman suffering from chronic headache, and with a history of mental disorders, who ‘treated’ herself with bee stings.

## Case presentation

A female, 50-year-old, anorectic (BMI 15.9) inpatient was referred to the ward of the pain unit of our university clinic with a long history of different pain syndromes (average pain intensity score on ´Numeric Rating Scale´: 8/10; 0 = "no pain", 10 = "worst pain imaginable"), including "polyneuropathy". For many years, she complained about the chronic pain felt mainly in the cervical and lumbar spine. For about one year, she suffered from a chronic regional pain syndrome (CRPS-1) after a fall on the right hand without fracture. Furthermore, for two years, she suffered from a left-sided hypaesthesia without an organic correlate, and previously, she had a left-sided hypaesthesia for one year. Finally, the patient complained about a predominantly left-sided, episodic migraine with aura (ICD-10: G43.1; IHS-ICHD-2: 1.2) with about three to four attacks per month. For all pain syndromes so far, no medication achieved any relief, and many substances were not tolerated due to reported side effects. For her headache disorder, she initially started using daily bee stings on her back as a ‘prophylactic therapy’. Subsequently, she reported that the headaches completely stopped. At about the same time as starting the therapy with bee venom (BV), a pain in the left upper limb without an organic correlate occurred. During the inspection of the back, many scars could be found due to the repeated bee stings and supposed local skin infections. Lesions were identified especially on the right arm, such as from burning on the oven due to reduced pain sensation. Clinically, a hypaesthesia to blunt and sharp stimuli on the right side could be found. A cerebrospinal fluid puncture showed no abnormalities, particularly no inflammatory causes. Signs of a distinct rheumatic disease could not be found. A psychotherapeutic evaluation showed signs of a dissociative disorder (ICD-10: F44), anorexia (ICD-10: F50.0), and a somatoform pain syndrome (ICD-10: F45.4). Aspects of a borderline personality disorder (ICD-10: Z73.1) were also identified. Several past and actual psychosocial problems could be found, but the self-administered Patient Health Questionnaire (PHQ) [2] showed no pathological findings. Neurological or inflammatory reasons for the hemihypaesthesia could not be found and were rated as part of psychic disease. Thus, we diagnosed primarily a somatoform pain disorder (ICD-10: F45.4) with many different somatic aspects. The suspended migraine attacks by the BV therapy were, in our opinion, due to placebo effects and part of the patient’s self-harming behaviour. For the neuropathic pain, pregabalin, due to severe lactose intolerance as a solution, was titrated up to a dose of 60 mg twice daily and was tolerated. Psychotherapeutic (cognitive behavioural therapy, CBT) co-treatment focused on diagnostic aspects, conveying coping strategies and motivation for ambulatory psychotherapy. The patient was advised to stop the BV therapy, as this kind of treatment lacks any evidence and inflammatory or other adverse effects that may further harm the patient could not be excluded. The patient could not accept any psychological explanations or (co)diagnoses, and she refused further exploration during the hospital treatment. She could benefit only in aspects of pain coping. Nevertheless, she seemed motivated to start ambulatory psychotherapy. The effect of the started pregabalin therapy is not known, since after discharge no further treatment or information could be gained. Therefore, no data about further BV therapy can be presented.

## Discussion

In this case report, we presented the case of a middle-aged woman suffering from chronic headache and with a history of mental disorders who ‘treated’ herself with bee stings. In our judgement, it has to be assumed that the suspended migraine attacks by the BV therapy were due to placebo effects and part of the patient’s self-harming behaviour. The use of BV (Apis mellifera) is propagandized by so-called apitherapy, a branch of complementary and alternative medicine. Up to 140 μg of venom is released per bee sting [3]. In Wikipedia (June 24, 2017) one can read: ‘The more modern study of apitherapy, specifically bee venom, was initiated through the efforts of Austrian physician Phillip Terc in his published results "Report about a Peculiar Connection Between the Bee stings and Rheumatism" in 1888. More recent popularity can be attributed to the Hungarian physician Bodog F. Beck who coined the term "bee venom therapy", and to Charles Mraz (1905–1999), a beekeeper from Vermont, United States over the past 60 years.’ Melittin, a basic 26-amino-acid polypeptide that constitutes 40–60% of dry BV, seems to be the major pain-producing substance of BV, by which peripheral persistent pain and hyperalgesia (or allodynia), primary nociceptive neuronal sensitization, and CNS synaptic plasticity (or metaplasticity) can be readily induced [4]. Occasionally, sensational articles in the lay press have reported about the self-application of bee stings in some known celebrities (e.g., http://www.huffingtonpost.in/entry/gwyneth-paltrow-apitherapy-bee-stings_us_5702b0dde4b083f5c6084d59), which could induce a new kind of fashionable trend. With regard to pain, most published data come from bee venom acupuncture (BVA), which involves injecting diluted BV into acupoints. It is used, for example, for arthritis, pain, and rheumatoid diseases, especially in the treatment of central post-stroke pain or post-stroke shoulder pain and, in combination with physiotherapy, in the treatment of adhesive capsulitis. Additionally, recent clinical and experimental studies have demonstrated the application of BV and BV-derived active components to a wide range of immunological, inflammatory, and neurodegenerative diseases, including Parkinson's disease and chemotherapy-induced peripheral neuropathy. Moreover, one should stress the fact that, on the one hand, the concept of ‘pain against pain’ or (controlled) ‘anti-pain’ has its advocates, and, on the other hand, just the use of painful bee stings could be a symptom of disorders in body awareness [5]; non-suicidal self-injury, which could be found especially in eating [6-7]; and/or cluster B personality disorders with their dysfunctional patterns of emotional/affective regulation [8]. For now, one must conclude that therapeutic BV injection may be beneficial for some patients (cave: mastocytosis and/or elevated baseline serum tryptase concentration, severe cardiovascular disease), but may also be — aside from (local) toxic, (IgE-mediated systemic) allergic, or anaphylactic reactions — very harmful [9]. Moreover, all pain dimensions [10] and bio-psycho-social aspects a patient presents must be considered.

## Conclusions

BV is widely used in complementary medicine, despite the lack of evidence-based knowledge. ‘Pain against pain’ could be subjectively beneficial in individual patients and could represent a self-harming behaviour that might be a symptom of a mental disorder.
